# Reactivation of *Tert* in the medial prefrontal cortex and hippocampus rescues aggression and depression of Tert^−/−^ mice

**DOI:** 10.1038/tp.2016.106

**Published:** 2016-06-14

**Authors:** Q-G Zhou, H-Y Wu, H Zhou, M-Y Liu, H-W Lee, X Liu, S Devkota, E J Ro, D-Y Zhu, H Suh

**Affiliations:** 1 Department of Stem Cell Biology and Regenerative Medicine, Lerner Research Institute, Cleveland Clinic, Cleveland, OH, USA; 2 Department of Pharmacology, Pharmacy College, Nanjing Medical University, Nanjing, China; 3 Department of Biochemistry, College of Life Science and Biotechnology, Yonsei Laboratory Animal Research Center, Yonsei University, Seoul, Korea; 4 Department of Chinese Medicine, College of Pharmacy, Nanjing University of Chinese Medicine, Nanjing, China

## Abstract

The role of telomerase reverse transcriptase (TERT) has been extensively investigated in the contexts of aging and cancer. Interestingly, *Tert*^−*/*−^ mice exhibit additional but unexpected aggressive and depressive behaviors, implying the potential involvement of TERT function in mood control. Our conditional rescue experiments revealed that the depressive and aggressive behaviors of *Tert*^−*/*−^ mice originate from *Tert* deficiency in two distinct brain structures. Reactivation of *Tert* in the hippocampus was sufficient to normalize the depressive but not the aggressive behaviors of *Tert*^−*/*−^ mice. Conversely, re-expression of *Tert* in the medial prefrontal cortex (mPFC) reversed the aggressive but not the depressive behavior of *Tert*^−*/*−^ mice. Mechanistically, decreased serotonergic signaling and increased nitric oxide (NO) transmission in the hippocampus transduced *Tert* deficiency into depression as evidenced by our observation that the infusion of a pharmacological agonist for serotonin receptor 1a (*5-*HTR1A) and a selective antagonist for neuronal NO synthase into the hippocampus successfully normalized the depressive behavior of *Tert*^−*/*−^ mice. In addition, increased serotonergic transmission by the *5-*HTR1A agonist in the mPFC was sufficient to rescue the aggressive behavior of *Tert*^−*/*−^ mice. Thus, our studies revealed a novel function of TERT in the pathology of depression and aggression in a brain structure-specific manner, providing direct evidence for the contribution of TERT to emotional control.

## Introduction

Telomerase is a ribonucleoprotein complex that adds 6-bp DNA repeat sequences to chromosome ends, which are referred to as telomeres, and thereby prevents the telomeres from shortening during mitosis.^1, 2^ Telomerase consists of two highly conserved protein and RNA components: telomerase reverse transcriptase (TERT) is the core catalytic protein component that contains a cellular reverse transcriptase domain, and the RNA component, *Terc*, serves as a template for telomere synthesis.^3^ Because DNA polymerase cannot replicate chromosomes completely to the DNA termini, telomeres shorten as cell division proceeds. Most post-mitotic cells have low to undetectable telomerase activity. This observation raised the possibility that telomere length functions as a 'molecular clock', where shortened telomeres accumulated over successive cell divisions limit the proliferative capacity of progenitors, progressively leading to cellular senescence.^2, 4^

Consistent with this hypothesis, *Tert*-deficient mice exhibited accelerated aging processes, and reactivation of telomerase was sufficient to reverse the age-related decline in many organs of *Tert*^−*/*−^ mice.^5, 6^ Upregulated telomerase activity, however, has been found in many cancers, and overexpression of telomerase extended the lifespan of cells.^7, 8, 9^ These observations emphasize that telomerase activity is critical for the function of actively proliferating cells and thus should be tightly regulated;^10^ high levels of telomerase activity may lead to the unregulated proliferation observed in cancer, while low levels of telomerase activity may accelerate the cellular aging process.

In addition to the role of telomerase in cancer and aging, telomerase abnormalities in the hematological system have also been reported in patients with mood-related disorders. Decreased telomerase activity and accelerated telomere shortening in leukocytes has been identified in individuals with chronic stress,^11^ major depressive disorders^12^ and schizophrenia.^13^ Although animal studies are thus far limited, a potential involvement of telomerase in mood disorders has also been suggested. Elevated anxiety-like behaviors were observed in *Tert*-deficient mice.^14^ Our previous study also showed that systematic inhibition of telomerase activity induced depressive behavior in the mouse.^15^ However, the anatomical regions involved and molecular mechanisms that link *Tert* deficiency to psychiatric deficits have remained undetermined.

Here, using *Tert*^−*/*−^ mice,^16^ we investigated how TERT regulates mood-related behavior. Our behavioral analyses uncovered a novel role of TERT in the control of aggressive and depressive behavior. Using a tissue-specific, conditional rescue strategy, we revealed that re-expression of *Tert* in the medial prefrontal cortex (mPFC) reversed the aggressive behaviors of *Tert*^−*/*−^ mice, whereas reactivation of *Tert* in the hippocampus was sufficient to rescue the depressive behaviors of these mice. Molecularly, dysregulated transmission of serotonergic and nitric oxide (NO) signals in the hippocampus and mPFC was evident in *Tert*^−*/*−^ mice. In our rescue experiments, re-expression of *Tert* in the hippocampus and mPFC normalized the altered expression levels of serotonin receptor 1a (5-HTR1A) and neuronal NO synthase (nNOS), which correlated with the recovery of the respective depressive and aggressive behaviors of *Tert*^−*/*−^ mice. Moreover, pharmacological activation of 5-HTR1A or inhibition of neuronal nNOS in the hippocampus successfully normalized the depressive behavior of *Tert*^−*/*−^ mice, while treatment with a 5-HTR1A agonist in the mPFC rescued the aggressive behavior of *Tert*^−*/*−^ mice. Thus, our study clearly demonstrated that the mPFC and hippocampus are two distinct brain areas that transduce *Tert* deficiency into aggression and depression and that 5-HTR1A and nNOS mediate the effect of *Tert* on emotional stability.

## Materials and methods

### Mice

All animal procedures were approved by the Institutional Animal Care and Use Committee of the Cleveland Clinic and Nanjing Medical University. Mice were housed in a temperature- and humidity-controlled environment with an alternating 12-h light/dark cycle.

The production of mTERT knockout mice (*Tert*^−*/*−^) and the lack of telomerase activity in these mice were previously described.^16^ *Tert*^−*/*−^mice were backcrossed to FVB/N mice to produce mice heterozygous for *Tert*. *Tert*^*+/*−^ mice were intercrossed to produce the first generation of *Tert*^−*/*−^ mice (F1). All subjects in the experiments were from the fourth or fifth generation after intercrossing F1 *Tert*^−*/*−^ homozygotes. Two- to 3-month-old male mice were used for behavioral tests.

### Construction of LV-mTert–EGFP

*mTer*t complementary DNA was prepared by reverse transcription–PCR from an mRNA library of mouse embryonic stem cells. The primer sequences were as follows: forward, 5′-GTAGAACGCAGATCGAATTCATGACCCGCGCTCCTCG-3′ reverse, 5′-CCCTTGCTCACCATGAATTCGTCCAAAATGGTCTGAAAGTC-3′. The PCR fragment corresponding to *Tert* complementary DNA was digested with *Bam*HI and *Age*I and ligated into downstream of a ubiquitous Ubi promoter in a lentiviral vector (Genechem company, Shanghai, China). A reporter gene expressing a enhanced green fluorescent protein (EGFP) was also expressed under the control of the SV40 promoter (pUbi-mTert-pSV40–EGFP). The production of lentivirus (LV) was previously described.^17^ Briefly, using Lipofectamine 2000 (Invitrogen, Carlsbad, CA, USA), 20 μg of pUbi-mTert-pSV40–EGFP, 10 μg of pCMV-VSVG (vesicular stomatitis virus glycoprotein), 7.5 μg of pRSV-REV (lentiviral packaging plasmid) and 3.5 μg of pMDLg/pRRE (lentiviral packaging plasmid) were cotransfected into 293 T cells. After 48 h, the supernatant was harvested from the 293 T cells, filtered at 0.45 μm and pelleted by ultracentrifugation at 18 000*g* for 2 h at 4 °C (~2 × 10^9^ transducing units per ml). A LV expressing EGFP alone (LV–EGFP) was also produced and used as a control.

### Stereotactic injection

The detailed procedures regarding stereotactic surgery and injection were previously described.^17^ Briefly, adult mice were anesthetized with a mixture of ketamine (100 mg kg^−1^, ACE Surgical Supply, Brockton, MA, USA) and xylazine (10 mg kg^−1^, Sigma-Aldrich, St Louis, MO, USA) and placed in a stereotactic apparatus (David Kopf Instruments, Tujunga, CA, USA). LVs or drugs such as 7-nitroindazole (7-NI, 10 μm, Sigma-Aldrich) or 8-hydroxy-2-dipropylaminotetralin hydrobromide (8-OH-DPAT, 5 μm, Sigma-Aldrich) were stereotactically delivered into both sides of the dentate gyrus (DG) of the hippocampus (2 μl; coordinates: 2.3 mm posterior to the bregma, 1.35 mm lateral to the midline and 2.3 mm below the dura)^15^ or the mPFC region (2 μl; coordinates: 1.8 mm anterior to the bregma, 0.8 mm lateral to the midline and 1 mm below the dura).^18^ The mice were recovered on a hot pad (37 °C) and returned back to their home cages.

### Western analysis

Procedures for western analysis were previously described.^19^ The hippocampus and mPFC were homogenized in sample buffer containing 200 mm Tris-buffered saline, 4% SDS, 20% glycerol and 10% 2-mercaptoethanol, and denatured by boiling for 5 min. Primary antibodies used were as follows: nNOS (rabbit, 1:200; Zymed Laboratories, San Francisco, CA, USA), 5-HTR1A (rabbit, 1:100; Bioss, Bejing, China), GAPDH (rabbit, 1:2000; Sigma, St. Louis, MO, USA) and FLAG (rabbit, 1:500; Enzo, Farmingdale, NY, USA). Horseradish peroxidase-conjugated goat anti-rabbit antibody was used as a secondary antibody. A volume of 10 μl lysate containing 10 μg protein was loaded for each sample. Enhanced chemiluminescence (Pierce, Carlsbad, CA, USA) was used to detect the signals.

### Behavioral measures

#### Resident–intruder paradigm

An adult male *nNOS*^−*/*−^ mouse, a strain that has been reported to show elevated aggressive behavior, was used as an intruder.^20^ An intruder mouse was transferred into the residents' cage (four males per cage), and attack episodes by the resident mice were recorded for 15 min. The intruder was typically attacked and defeated by the residents and showed freezing submissive behavior. Aggressive behaviors of the resident mice were scored when they bit, attacked laterally and displayed piloerection. The latency to the first attack bite, the frequency of bites and the duration of attack episodes were manually quantified. Data collected from four wild-type (WT) or *Tert* ^−*/*−^ mice in the same resident cage were analyzed as a unit.

#### Offensive aggressive behavior test

Four adult WT or *Tert*^−*/*−^ mice were simultaneously introduced into a clear glass aquarium (38.5 × 26.5 × 30.7 cm). The latency to the first biting attack, the total number of attack bites and the total duration of attack episodes during 15 min were recorded and analyzed. Data collected from four WT or *Tert* ^−*/*−^ mice in the same resident cage were analyzed as a unit.^20^

#### Tail suspension test

A mouse was suspended from its tail for 6 min in a tail suspension chamber (Hamilton Kinder TS100, Kinder Scientific, Poway, CA, USA).^19^ The frequency and duration of escape behaviors were recorded during tail suspendsion test (TST). Immobility, which was defined as the cessation of all movements during a 6-min test period, was calculated to measure depressive status. Data from the mice that dropped from the hook, climbed up to the hook and struggled dramatically the entire time were excluded.

#### Forced swim test

Mice were individually forced to swim in an open cylindrical container (diameter, 28 cm; height, 33 cm) containing 20 cm of water (25±1 °C), and the duration of immobility during a 6-min period was measured.^21^ Immobility was scored when a mouse ceased struggling and remained floating motionlessly. Water was replaced between trials. Following swim sessions, mice were placed underneath a heating lamp for ~30 min before they were returned to their home cages. Forced swim test (FST) sessions were videotaped (Motor-Monitor System SF16R, Kinder Scientific), and immobility was measured in a double-blinded manner. Data from the mice that sunk into the water, climbed up to the container or floated the entire time were excluded.

#### Sucrose preference test

The sucrose preference test was performed as previously described.^22^ Food and water were removed from cages for 20 h. Two bottles containing water or 1% (W/V) sucrose water were presented in the cage simultaneously, and animals had free access to these bottles for the next 10 h. Sucrose preference was scored as the ratio of the intake of 1% sucrose water to total water intake, which included that of both 1% sucrose water and regular water.

#### Elevated maze test

The apparatus for the elevated maze test (EMT) consisted of two open arms (30 × 5 cm), two enclosed arms (30 × 5 cm, with the end and side walls 15 cm high) and a connecting central platform (5 × 5 cm). The maze was raised 38.5 cm above the floor. Each mouse was placed on the central platform facing an open arm and allowed to freely explore for 5 min. An arm entry was scored when all four legs completely entered the maze arm. Data from the mice that dropped from the maze and stayed in one place the entire time were excluded.

#### Light–dark test

The dark–light box contained two plastic chambers connected by a small tunnel. A dark chamber (20  × 15 cm) was covered, and the walls of the light chamber (30 cm × 15 cm) were transparent and illuminated by a light located above (600 lx). A mouse was placed in the dark compartment, and the latency to the first exit, the number of exits from the dark chamber and the total time spent in the light compartment were recorded for 5 min.

#### Open field test

The test arena consisted of a bottom plastic plate (56.13–56.13 cm) and four surrounding plastic walls (35.18 cm high). The bottom plate was divided into 256 squares, and each square was marked with visible lines. A mouse was placed on a corner square of the arena, facing the corner and allowed to freely explore the open field for 5 min. The arena was cleaned with 70% EtOH between trials. Mobility was scored when a mouse crossed a border with both hind limbs. The number of square crossings and the frequency of standing during 5 min were measured.

### NO measurement

NO content in the hippocampus or prefrontal cortex was determined as previously described.^22^ The samples were homogenized in 10 vol of deionized water and centrifuged at 1000*g* for 15 min at 4 °C. NO*_x_* content was measured in the supernatants using a commercially available kit (Jiancheng Bioengineering, Nanjing, China) and is expressed as nmol per mg protein.

### 5-HT ELISA

The concentration of serotonin in the plasma was determined following the manufacturer's instructions (Serotonin ELISA Kit, Abcam, Cambridge, UK). Each test measured samples in triplicate, and a standard curve was plotted each time to ensure the validity of the assay.

### Immunohistochemistry

The mice were anesthetized with a mixture of ketamine (100 mg kg^−1^) and xylazine (10 mg kg^−1^) and perfused transcardially with saline followed by 4% paraformaldehyde. Brains were removed and postfixed overnight in the same solution. To identify the cell types, labeling was carried out on 40-μm free-floating sections as described. Primary antibodies were as follows: nNOS (rabbit, 1:200), 5-HTR1A (rabbit 1:100), NeuN (mice, 1:200; Millipore, Billerica, MA, USA) and GFAP (chicken, 1:1000; Abcam, Cambridge, MA, USA). They were prepared in 0.1 m PBS with 3% goat serum and 0.3% Triton X-100, and visualized with a Cy3-conjugated secondary antibody (1:200; Thermo Fisher Scientific, Waltham, MA, USA). Nuclei were visualized with 4′-6-diaminodino-2-phenylindole (DAPI, Sigma-Aldrich). Every twelfth section throughout the hippocampus was processed for nNOS or 5-HTR1A immunohistochemistry and counting. Two sections containing mPFC were processed for analysis of nNOS or 5-HTR1A in the mPFC.

### Statistics

Comparisons among multiple groups were performed using one-way analysis of variance followed by Scheffe's *post hoc* test. Comparisons between two groups were performed with the two-tailed Student's *t*-test. Data are presented as mean±s.e.m.; *P<*0.05 was considered statistically significant. Detailed statistical analyses regarding the experiments performed in Figures 2 and 4 are described in Supplementary Tables 1 and 2. This study complies with randomization. The investigator was blinded to the group allocation during the experiment and/or when assessing the outcome.

## Results

### Aggressive and depressive behaviors in *Tert*^−*/*−^ mice

**Figure 1:**
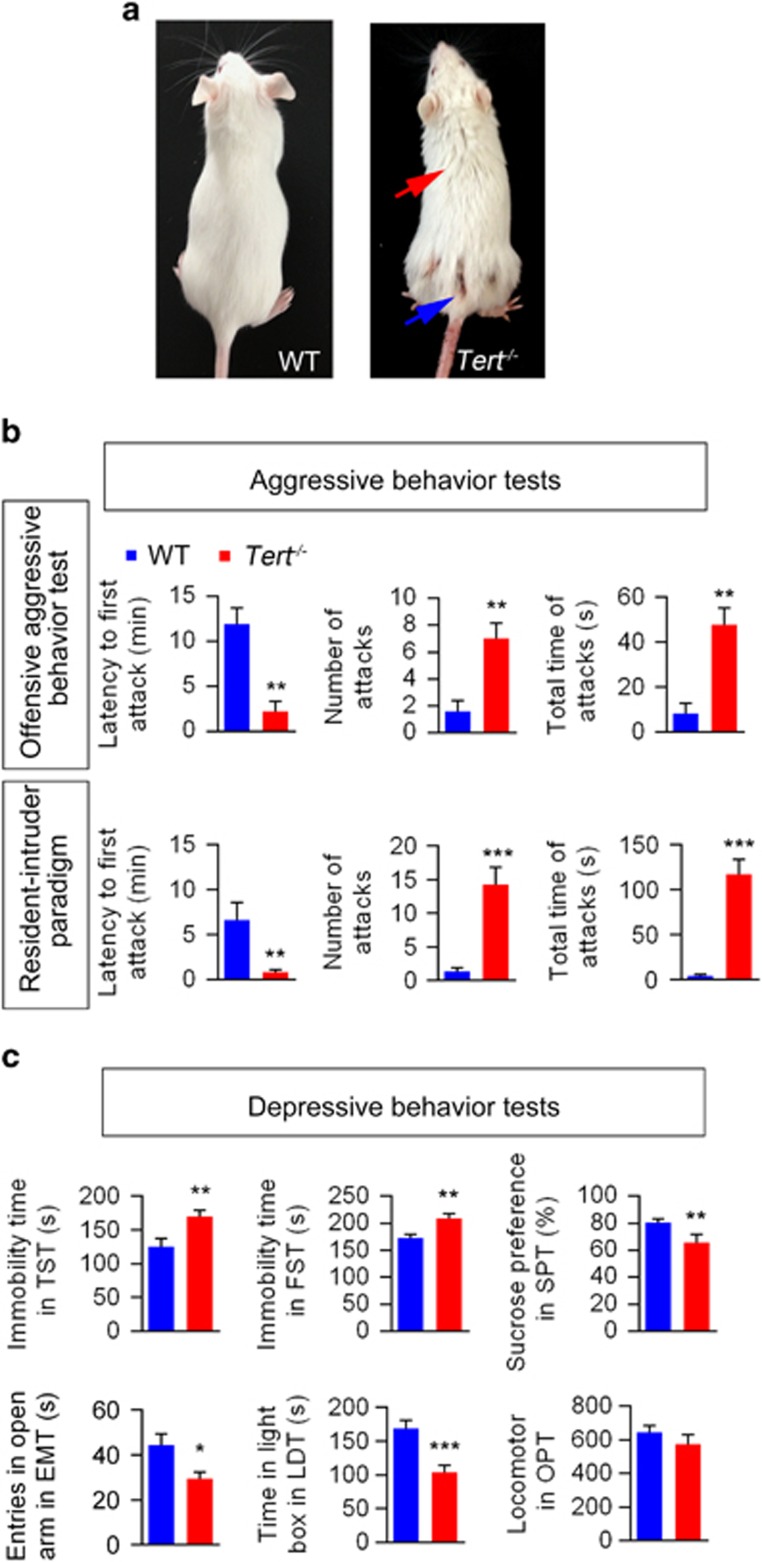
Aggressive and depressive phenotype of *Tert*^−*/*−^ mice. (**a**) A representative photo of subordinate *Tert*^−*/*−^ mice. The blue arrow indicates areas of the coat where the animal has been bitten. The red arrow indicates scruffy areas of poor coat quality. (**b**) Top panel (offensive aggressive behavior): the latency to the first biting attack (left), the total number of attacks (middle) and the total duration of attack episodes (right) are presented. Bottom panel (resident–intruder paradigm): the latency to the first biting attack (left), the total number of attacks (middle) and the total duration of attack episodes initiated by the resident (right) are presented. (**c**) Immobility time in the TST and the FST and sucrose preference in the SPT showed the depressive behavior of *Tert*^−*/*−^ mice. In addition, the total time of mouse entry into the open arms in the EMT and the time spent in the light box in the LDT were examined. The locomotion of mice was recorded in the OPT. *n*=20. Data represent the mean±s.e.m. *P<0.05; **P<0.01; and ***P<0.001 by two-way *t*-test compared with WT mice. EMT, elevated maze test; FST, forced swim test; LDT, light–dark test; SPT, sucrose preference test; TST, tail suspension test; WT, wild type.

To investigate the potential relationship between telomerase and mood disorders, *Tert*^−*/*−^ mice in which telomerase activity is deficient were examined.^16^ *Tert*^−*/*−^ mice exhibited bite wounds and scruffy coats, suggesting the presence of aggressive behavior and depression in these mice (Figure 1a).^23^

In an offensive aggressive behavior test,^20^ *Tert*^−/−^ mice exhibited a significantly shorter latency to the first biting attack, increased attack frequency and a longer total duration of attack episodes compared with WT mice (*P*=0.0035, *P*=0.0048 and *P*=0.0018, respectively, by *t*-test; *n*=20 for each group; Figure 1b). The resident–intruder paradigm test, a standard test used to measure aggressive behavior in rodents, also confirmed the aggressive phenotype of *Tert*^−/−^ mice.^20, 24^ Consistent with the results of the offensive aggression test, in the resident–intruder paradigm test *Tert*^−*/*−^ mice showed aggressive behavior, as supported by a significantly shorter latency to the first biting attack, a higher number of attacks and a longer total attack time (*P*=0.0094, *P*=0.0002 and *P*=0.0001, respectively, by *t*-test; *n*=30 for each group; Figure 1b).

We used a series of behavioral tests to measure different endophenotypes of depression: behavioral despair, anhedonia and anxiety-related behaviors. To evaluate behavioral despair, the TST and the FST were used.^15, 22^ Compared with WT mice, *Tert*^−/−^ mice exhibited a significantly prolonged immobility time in the TST (*P*=0.0096, *t*-test; WT, *n*=20; *Tert*^−/−^ mice, *n*=17) and the FST (*P*=0.0027, *t*-test; WT, *n*=20; *Tert*^−/−^ mice, *n*=18; Figure 1c), revealing that *Tert*^−/−^ mice quickly abandoned attempts to escape from an adverse stimulus and thus exhibited depressive-like symptoms. *Tert*^−/−^ mice also showed decreased sucrose preference, suggesting a reduced ability to experience pleasure (*P*=0.0162, *t*-test; WT, *n*=18; *Tert*^−/−^ mice, *n*=13) (Figure 1c). We also measured anxiety-related behavior by performing an EMT and a light–dark test.^25, 26^ *Tert*^−*/*−^ mice showed decreased entry time in the open arm in the EMT (*P*=0.0177, *t*-test; WT, *n*=19; *Tert*^−/−^ mice, *n*=17) and in the light box in the light-dark test (*P*=0.0003, *t*-test; WT, *n*=18; *Tert*^−/−^ mice, *n*=16), indicating that *Tert*^−/−^ mice displayed elevated anxiety compared with WT mice (Figure 1c). However, the locomotor ability of *Tert*^−/−^ mice did not change (Figure 1c, *P*=0.2783, *t*-test; WT, *n*=20; *Tert*^−/−^ mice, *n*=20), suggesting that behavioral changes were not due to altered motor functions.

### Re-expression of *Tert* in the hippocampus and mPFC rescued depression and aggression in *Tert*^−*/*−^ mice

**Figure 2:**
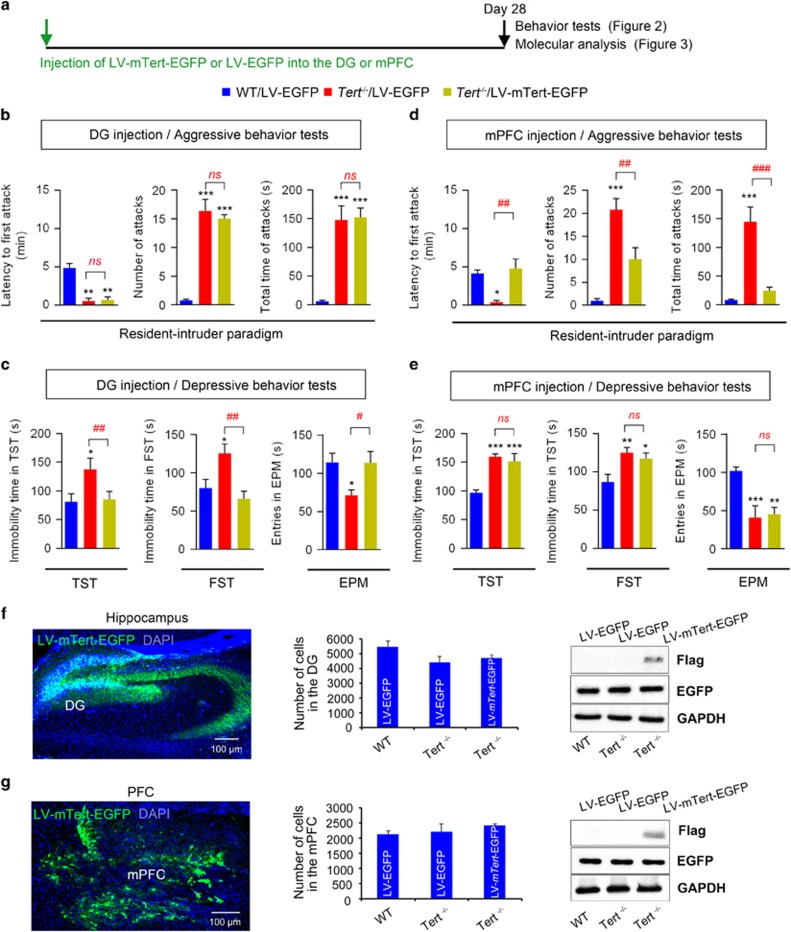
Rescue of aggression and depression in *Tert*^−*/*−^ mice by re-expression of *Tert* in the mPFC and hippocampus. (**a**) Experimental scheme. (**b**) Resident–intruder paradigm: the latency to the first attack bite (left), the total number of attacks (middle) and the total duration of attack episodes initiated by the resident (right) showed that aggressive behaviors were not affected by *Tert* re-expression in the DG. (**c**) Immobility time in the TST and the FST and the total time spent in the open arms in the EMT showed the rescue of the depressive phenotype of *Tert*^−*/*−^ mice by re-expression of *Tert* in the DG of the hippocampus. (**d**) Resident–intruder paradigm: the latency to the first attack bite (left), the total number of attacks (middle) and the total duration of attack episodes initiated by the resident (right) showed the reversal of aggressive behavior in *Tert*^−*/*−^ mice by re-expression of *Tert* in the mPFC. (**e**) Immobility time in the TST and the FST and the total time spent in the open arms of the EMT showed no effect of *Tert* re-expression in the mPFC on the depressive behavior of *Tert*^−*/*−^ mice. (**f**, **g**) The efficient transgene expression and viral coverage in the hippocampus and mPFC are shown by EGFP IHC (left), counting of EGFP-positive cells (middle) and western blot (right). *'*'*n'*' in each group is listed in Supplementary Table 1. Data represent the mean±s.e.m. One-way ANOVA, *P<0.05; **P<0.01; and ***P<0.001 compared with WT mice; ^#^*P<*0.05; ^##^*P<*0.01; and ^###^*P<*0.01 compared with *Tert* ^−/−^/LV–GFP mice; NS indicates no significance compared with *Tert* ^−/−^/LV–GFP. ANOVA, analysis of variance; DG, dentate gyrus; EGFP, enhanced green fluorescent protein; EMT, elevated maze test; FST, forced swim test; IHC, immunohistochemistry; mPFC, medial prefrontal cortex.

**Figure 3:**
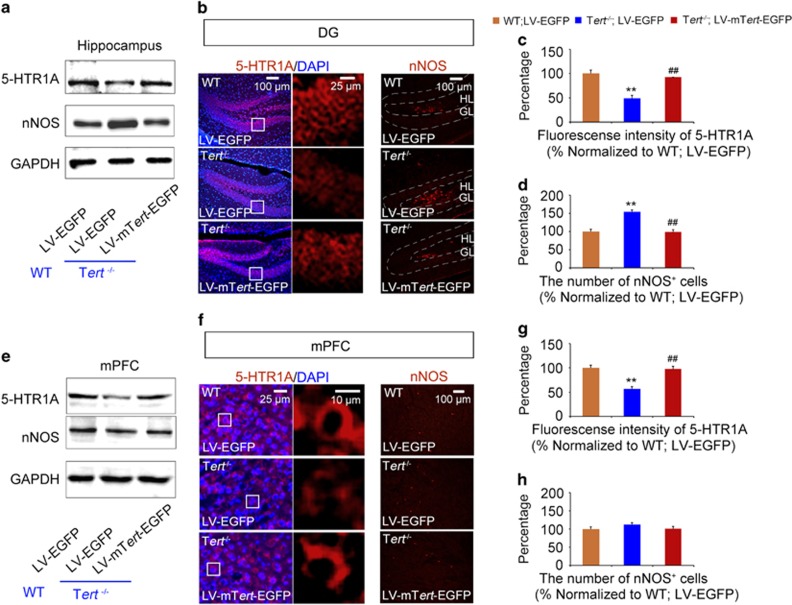
TERT regulates the expression of 5-HTR1A and nNOS in the mPFC and hippocampus. (**a**) Western blot showing the re-expression of TERT normalized the expression levels of 5-HTR1A and nNOS in the hippocampus of *Tert*^−*/*−^ mice. *n*=4. (**b**) IHC showed that the expression patterns of 5-HTR1A and nNOS in the hippocampus of *Tert*^−*/*−^ mice were rescued by re-expression of TERT. (**c**, **d**) Measurement of signal intensity and quantitative analyses show the normalization of 5-HTR1A and nNOS expression in the hippocampus of *Tert*^−*/*−^ mice by TERT re-expression. *n*=3. (**e**) Western blot showing that the re-expression of TERT normalized the expression level of 5-HTR1A in the mPFC of *Tert*^−*/*−^ mice. *n*=4. (**f**) IHC showed that the expression pattern of 5-HTR1A in the mPFC of *Tert*^−*/*−^ mice was rescued by re-expression of TERT. (**g**, **h**) Measurement of signal intensity and quantitative analyses show the normalization of 5-HTR1A and nNOS expression in the mPFC of *Tert*^−*/*−^ mice by TERT re-expression. *n*=3. Data represent the mean±s.e.m. One-way ANOVA, **P<0.01 compared with WT; LV–EGFP mice; ^##^*P<*0.01 compared with *Tert*^−*/*−^; LV–EGFP mice. ANOVA, analysis of variance; DG, dentate gyrus; EGFP, enhanced green fluorescent protein; IHC, immunohistochemistry; LV, lentivirus; mPFC, medial prefrontal cortex; TERT, telomerase reverse transcriptase.

Both the hippocampus and mPFC play a critical role in depressive and aggressive behaviors.^27, 28^ We tested whether re-expression of *Tert* in the hippocampus and mPFC was able to rescue the depression and aggression of *Tert*^−/−^ mice (Figure 2a). To achieve this goal, LV expressing a full-length mouse *Tert* complementary DNA and an EGFP reporter gene (LV-*mTert*–EGFP) was injected into both sides of either the DG of the hippocampus or the mPFC of *Tert*^−/−^ mice (*Tert*^−/−^/LV-*mTert*–EGFP). As a control, LV expressing only EGFP (but not *mTert*) was injected into WT (WT/LV–EGFP) and *Tert*^−/−^ (*Tert*^−/−^/LV–EGFP) mice. Twenty eight days later, the mice were subjected to behavioral tests (Figure 2) and molecular analyses (Figure 3).

*Tert* expression in the DG of the hippocampus efficiently rescued the depressive but not the aggressive behavior of *Tert*^−/−^ mice (Figures 2b and c). Re-expression of *Tert* in the hippocampus reversed the immobility time of *Tert*^−/−^ mice in the TST and the FST and also rescued the entry time in the open arm of the EMT in a statistically significant manner (Figure 2c, Supplementary Table 1). However, the latency to the first biting attack, the number of attack episodes and the total time of attack episodes remained unchanged when evaluated both by the offensive aggressive behavior test (Supplementary Figure 1a) and by the resident–intruder paradigm test (Figure 2b, Supplementary Table 1).

*Tert* expression in the mPFC successfully rescued the aggressive phenotypes of *Tert*^−*/*−^ mice. Re-expression of *Tert* in the mPFC restored the latency to the first biting attack, reduced the number of total attacks and decreased the total time of attack episodes during the resident–intruder paradigm test (Figure 2d, Supplementary Table 1) and during the offensive aggressive behavior test (Supplementary Figure 1b). However, re-expressing *Tert* in the mPFC did not reverse the depressive phenotype of *Tert*^−*/*−^ mice. The immobility time in the TST and FST and the entry time in the open arm in the EMT did not change in *Tert*^−*/*−^ mice (Figure 2e, Supplementary Table 1). Together, these data indicated that *Tert* plays a critical role in the hippocampus in the control of depressive behavior, while *Tert* expression in the mPFC controls aggressive behavior.

Using an EGFP reporter gene present in both the control and *mTert*-expressing lentiviral vectors, the efficiency and coverage of viral infection were examined. Western analysis of virus-infected brains showed that an equivalent amount of transgene was transduced by both the control and *mTert*-expressing LVs and that *TERT* was efficiently expressed (Figures 2f and g, Supplementary Figure 2). Histological analyses of EGFP-expressing cells revealed the efficient infection of both control and *mTert*-expressing virus into the DG of the hippocampus or mPFC. Infected virus spread throughout the target structures, covering a large volume of the DG or mPFC (Supplementary Figure 3). Importantly, the number of cells infected by control or *mTert*-expressing LV was comparable among the experimental groups, suggesting that the quality and injection of virus into the target areas were consistent across animal groups (Figures 2f and g).

### Expression of 5-HTR1A and nNOS is regulated by TERT

It has been shown that serotonin (5-hydroxytryptamine (5-HT)) and NO play a critical role in depression and aggression.^19, 22, 29, 30^ Several key components necessary for 5-HT and NO signaling are expressed in the hippocampus, as well as in the mPFC. Among the different types of 5-HT receptors, 5-HTR1A has been implicated in depression and aggression.^31, 32, 33, 34^ 5-HTR1A is expressed in neurons located in the DG of the hippocampus, as well as in the mPFC (Supplementary Figures 4a and b; Figures 3b and g). nNOS, a key enzyme essential for the production of NO, is also expressed in the hippocampus and mPFC (Supplementary Figures 4c–d). Interestingly, the expression of nNOS in the hippocampus is restricted to neurons in the hilus (Supplementary Figure 4c; Figure 3b). To determine whether serotonin or NO signaling is altered in *Tert*^−*/*−^ mice and whether tissue-specific reactivation of *TERT* restores altered serotonin or NO transmission, we examined the level of 5-HT and its cognate receptor, 5-HTR1A, as well as NO and nNOS in the hippocampus and mPFC of WT (WT/LV–EGFP), *Tert* knockout (*Tert*^−*/*−^/LV–EGFP) and *Tert* rescue (*Tert*^−*/*−^/LV -mTert–EGFP) mice as described in Figure 2a.

In the hippocampus, transmission of both serotonergic and NO was disrupted in *Tert*^−*/*−^ mice. While the concentrations of 5-HT did not change (Supplementary Figure 5a), the amount of 5-HTR1A expressed in *Tert*^−*/*−^ mice was significantly reduced (Figure 3a). Subsequent immunohistochemistry confirmed the decreased expression of 5-HTR1A in neurons located in the DG of the hippocampus in *Tert*^−*/*−^ mice (Figures 3b and c). In contrast to the decreased serotonergic transmission, NO transmission increased in *Tert*^−*/*−^ mice. In *Tert*^−*/*−^ mice, the level of NO (Supplementary Figure 5b), the expression of nNOS protein, and the number of nNOS-expressing neurons significantly increased in the hilus of the hippocampus (Figures 3a, b and d). Surprisingly, re-expressing *Tert* in the hippocampus of *Tert*^−*/*−^ mice (*Tert*^−*/*−^/LV-mTert–EGFP) reversed the altered expression of 5-HTR1A and nNOS to normal levels compared with WT mice (WT/LV–EGFP; Figures 3a–d).

In the mPFC, only serotonergic transmission was impaired in *Tert*^−*/*−^ mice. Consistent with unchanged concentrations of both 5-HT and NO (Supplementary Figure 5b), the level of nNOS protein and the number of nNOS-expressing neurons was not altered in the mPFC of *Tert*^−*/*−^ mice (Figures 3e, f and h). However, the expression of 5-HTR1A was significantly reduced, as determined by western and immunohistochemistry analyses (Figures 3e, f and g). In the brains of *Tert*^−*/*−^ mice that received LV-*mTert*–EGFP, reactivation of TERT in the mPFC normalized the altered expression of 5-HTR1A, while the number of nNOS-expressing cells remained unchanged. These data collectively showed that the expression of 5-HTR1A and nNOS was disrupted in the brains of *Tert*^−*/*−^ mice and that reactivation of TERT in the brains of *Tert*^−*/*−^ mice normalized this altered expression, with concomitant recovery of the aggressive and depressive behavioral phenotype of *Tert*^−*/*−^ mice.

### Pharmacological normalization of 5-HT and NO transmission in the DG and mPFC rescues the behavioral abnormalities of *Tert*^−*/*−^ mice

**Figure 4:**
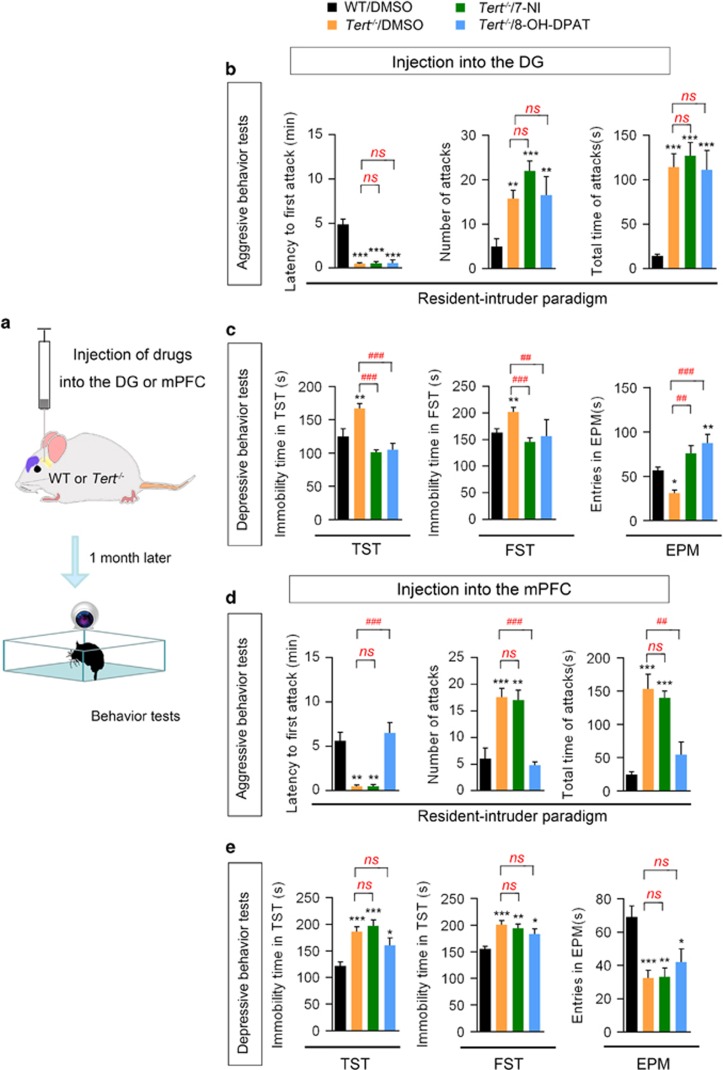
5**-**HTR1A and nNOS in the mPFC and hippocampus underlie the phenotype of *Tert*^−*/*−^ mice**.** (**a**) Diagram describing the design of the experiment. (**b**) Resident–intruder paradigm: the latency to the first attack bite (left), the total number of attacks (middle, and the total duration of attack episodes initiated by the resident (right) showed no effect of 7-NI or 8-OH-DPAT infusion in the DG of the hippocampus on aggression in *Tert*^−*/*−^ mice. (**c**) Immobility time in the TST and FST and the total time spent in the open arms in the EMT showed the rescue of the depressive behavior of *Tert*^−*/*−^ mice by infusion of 7-NI or 8-OH-DPAT into the DG. (**d**) Resident–intruder paradigm: the latency to the first attack bite (left), the total number of attacks (middle) and the total duration of attack episodes initiated by the resident (right) revealed the normalization of aggressive behavior of *Tert*^−*/*−^ mice by the infusion of 8-OH-DPAT into the mPFC. (**e**) Immobility time in the TST and the FST and the total time spent in the open arms in the EMT showed no effect of 7-NI or 8-OH-DPAT in the mPFC on depression in *Tert*^−*/*−^ mice. '*n*' in each group is listed in Supplementary Table 2. Data represent the mean±s.e.m. One-way ANOVA, *P<0.05; **P<0.01; and ***P<0.001 compared with WT/DMSO; ^##^*P<*0.01; ^###^*P<*0.001 compared with *Tert* ^−/−^/DMSO; NS indicates no significance compared with *Tert* ^−/−^/DMSO. ANOVA, analysis of variance; DG, dentate gyrus; EMT, elevated maze test; FST, forced swim test; mPFC, medial prefrontal cortex; TST, tail suspension test.

To determine the role of the altered levels of 5-HTR1A and nNOS in the behavioral abnormalities of *Tert*^−*/*−^ mice, we selectively increased or decreased the activity of 5-HTR1A and nNOS using the pharmacological drugs 8-OH-DPAT, a 5-HTR1A-selective agonist,^25^ or 7-NI, a selective nNOS antagonist.^22^ First, we administered 8-OH-DPAT (5 μm, 2 μl) or 7-NI (10 μm, 2 μl) into the DG bilaterally and performed behavioral tests 28 days later (Figure 4a). WT mice infused with DMSO were used as a control. The infusion of 8-OH-DPAT or 7-NI into the hippocampus significantly reduced immobility time in the TST and the FST and also increased entry time in the open arm in the EMT, indicating that either 5-HTR1A activation or nNOS inhibition in the hippocampus rescued the depressive phenotype of *Tert*^−*/*−^ mice (Figure 4c, Supplementary Table 2). However, 8-OH-DPAT or 7-NI infusion into the hippocampus did not affect aggressive behaviors, as shown by the resident–intruder paradigm test (Figure 4b, Supplementary Table 2). In *Tert*^−*/*−^ mice infused with 8-OH-DPAT or 7-NI, the latency to the first biting attack, the number of attacks and the total time of attack episodes did not change (Figure 4b, Supplementary Table 2).

Next, we infused 7-NI (10 μm, 2 μl) or 8-OH-DPAT (5 μm, 2 μl) into the mPFC and examined the behavioral changes of *Tert*^−*/*−^ mice (Figures 4d and e). Interestingly, only infusion of 8-OH-DPAT, but not infusion of 7-NI, normalized the aggressive phenotype of *Tert*^−*/*−^ mice (Figure 4d, Supplementary Table 2), showing a significantly decreased latency to the first attack, a reduced number attacks and a shortened total time of attacks (Figure 4d, Supplementary Table 2). However, infusion into the mPFC with either 8-OH-DPAT or 7-NI did not affect the depressive behaviors of *Tert*^−*/*−^ mice (Figure 4e, Supplementary Table 2), as assessed by the TST, the FST and the EMT. These results showed that in the hippocampus, increased activation of 5-HTR1A and decreased activity of nNOS successfully restored the depressive behavior of *Tert*^−*/*−^ mice, and that in the mPFC treatment with a 5-HTR1A agonist rescued the aggressive behavior of *Tert*^−*/*−^ mice.

## Discussion

The recent observations that leukocyte telomere shortening is associated with mental illness suggested the potential involvement of dysregulated telomerase function in the pathology of psychiatric disorders.^11, 35, 36, 37, 38^ However, direct evidence supporting the relation between telomerase function and mood disorders has remained absent. In this report, using mice deficient for *Tert*, a key enzyme for the maintenance of telomere length, we demonstrated the requirement of mouse telomerase for mood stability.

**Figure 5:**
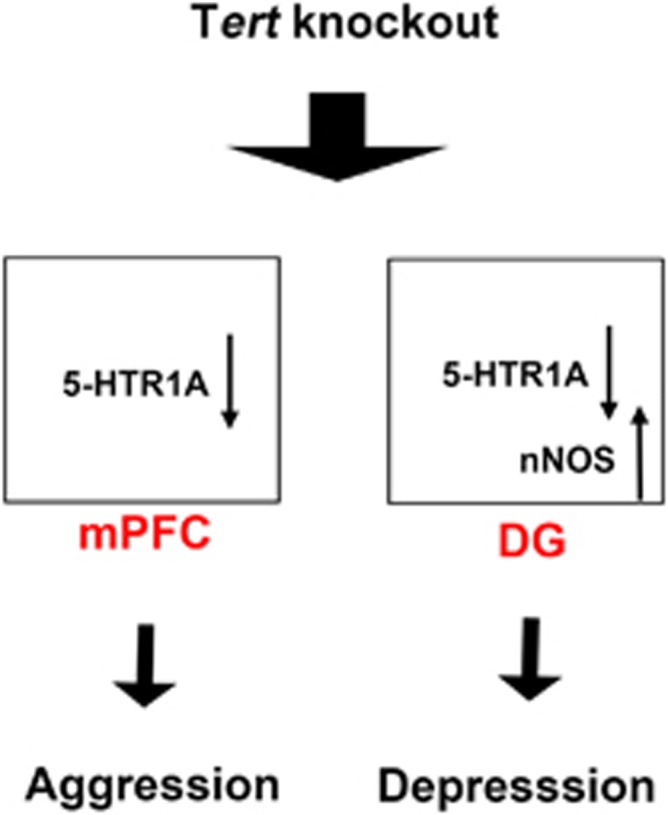
A model illustrating a molecular mechanism underlying the mood-related behaviors of *Tert*^−*/*−^ mice. A schematic model indicates that decreased serotonergic and/or increased NO transmission in the hippocampus account for the depressive behaviors of *Tert* ^−/−^mice and that decreased serotonergic signals in the mPFC lead to aggressive behaviors *Tert* ^−/−^mice. DG, dentate gyrus; mPFC, medial prefrontal cortex; NO, nitric oxide.

The function of TERT has been studied in the contexts of aging and cancer. However, we found that *Tert*^−*/*−^ mice exhibited additional but unexpected aggressive and depressive behavioral phenotypes. Our LV-mediated rescue experiments revealed that *Tert* controls aggression and depression by acting in different areas of the brain (Figure 5). Reactivation of *Tert* in the hippocampus was sufficient to normalize the depressive behavior of *Tert*^−*/*−^ mice, while the same treatment did not alter the aggressive behavior of *Tert*^−*/*−^ mice. Conversely, re-expression of *Tert* in the mPFC rescued the aggressive phenotype of *Tert*^−*/*−^ mice; however, the depressive phenotype of these mice was not affected. Thus, our study revealed a novel role of *Tert* in emotional control in a brain region-specific manner; the depressive and aggressive behaviors observed in *Tert*^−*/*−^ mice are anatomically distinct modalities, and *Tert* plays a critical role in depressive and aggressive behavior by acting in the hippocampus and mPFC, respectively (Figure 5).

In the hippocampus, the link between serotonergic and NO transmission and depression has been well-established.^22, 25^ Knockout mice lacking 5-HTR1A showed anxiety/depressive behaviors, while conditional expression of 5-HTR1A in the hippocampus and cortex successfully rescued the anxiety/depressive behavioral phenotypes of 5-HTR1A knockout mice.^39^ The requirement of hippocampal neurogenesis for the function of SSRI (selective serotonin reuptake inhibitor)-type antidepressants further supports the critical role of the hippocampus in the control of depressive behavior.^40^ In previous studies, we showed that increased nNOS expression in the hippocampus induced depressive behaviors, whereas a decreased level of nNOS functions as an antidepressant.^19, 22^ In the mPFC, serotonergic signaling has been also implicated in aggressive behaviors. Aggressive behaviors were shown to be associated with decreased 5-HT in the mPFC,^41^ whereas activation of 5-HTR1A in the mPFC reduced aggressive behavior.^42, 43, 44^ These results collectively suggested that decreased serotonergic and/or increased NO transmission in the hippocampus is sufficient to induce depressive behaviors and that decreased serotonergic signals in the mPFC lead to aggressive behaviors.

Our tissue-specific rescue experiments, as well as pharmacological epistasis studies demonstrated that 5-HTR1A and nNOS are downstream effector molecules that mediate *Tert*-dependent emotional behavior. In *Tert*^−*/*−^ mice, 5-HTR1A expression was decreased in both the hippocampus and mPFC, while nNOS expression was increased in the hippocampus, raising the possibility that disrupted serotonergic and NO signals may transduce the effect of *Tert* deficiency on depression and aggression. Consistent with this hypothesis, our LV-mediated rescue experiments showed that re-expression of *Tert* in the hippocampus or mPFC normalized the altered expression of 5-HTR1A and/or nNOS, which was associated with the recovery of depressive and aggressive behaviors of *Tert*^−*/*−^ mice. Moreover, epistatic analyses performed with pharmacological reagents that normalize the altered activity of 5-HTR1A or nNOS in *Tert*^−*/*−^ mice strongly suggested that 5-HTR1A and/or nNOS are downstream effector molecules that transduce TERT function to emotion. This pharmacological normalization of serotonergic and NO signaling reversed the behavioral deficits of *Tert*^−*/*−^ mice in a brain region-specific manner. Increased serotonergic transmission by infusion of a 5-HTR1A agonist, 8-OH-DPAT, or decreased NO signaling by infusion of an nNOS antagonist, 7-NI, into the hippocampus successfully reversed the depressive phenotype of *Tert*^−*/*−^ mice. In addition, 8-OH-DPAT infusion into the mPFC was sufficient to normalize the aggressive behavior of *Tert*^−*/*−^ mice. The combination of our tissue-specific rescue experiments and epistatic analyses using pharmacological reagents clearly demonstrated that *Tert* in the hippocampus and mPFC modulates depressive and aggressive behaviors through the serotonergic and NO pathways, respectively (Figure 5).

While our studies showed that serotonergic and/or NO signaling transduces the effect of TERT on mood control, whether TERT regulates emotion directly or indirectly is still speculative. It is possible that depression and aggression may be due to the accelerated cellular aging of *Tert*^−*/*−^ mice.^45^ Psychiatric alterations such as depression and aggression are known to be associated with Alzheimer's disease, an aging-dependent disorder.^46^ The correlation between telomere shortening and age-dependent processes has been well-established.^2^ Telomeres are a critical structure for the maintenance of genetic integrity by preventing chromosome ends from degrading. Because DNA polymerase fails to fully synthesize DNA terminal sequences (containing telomeres), the length of telomeres progressively shortens with successive cell divisions. To a certain extent, *Tert*, a cellular reverse transcriptase, has the ability to compensate for telomere loss by adding new telomere sequences onto chromosome ends; however, this is not sufficient to maintain telomere length as evidenced by the telomere shortening that occurs with age. Thus, the failure of telomere maintenance in *Tert*^−*/*−^ mice may accelerate the aging process, which in turn induces age-associated aggressive and depressive behaviors. Thus, our results can be interpreted to suggest that reactivation of TERT in the brain rescued the emotional deficits of *Tert*^−*/*−^ mice by reversing or slowing aging-associated processes.^5, 47, 48^ Alternatively, *Tert* may regulate emotional behavior in a telomere length- and elongation-independent manner.^49^ Recent studies proposed a non-canonical function for *Tert* as a transcriptional regulator. For example, it has been shown that *Tert* interacts with BRG1 in the β-catenin transcriptional complex and occupies the promoters of WNT-dependent genes, suggesting that *Tert* can act as a transcription factor.^50^ This observation raises the possibility that *Tert* may directly control the expression level of 5-HTR1A and/or nNOS. Future studies will dissect the direct molecular mechanism by which *Tert* controls emotional behavior.

## References

[bib1] Lundblad V, Wright WE. Telomeres and telomerase: a simple picture becomes complex. Cell 1996; 87: 369–375. 8898191 10.1016/s0092-8674(00)81358-6

[bib2] Armanios M, Blackburn EH. The telomere syndromes. Nat Rev Genet 2012; 13: 693–704. 22965356 10.1038/nrg3246 PMC3548426

[bib3] Autexier C, Lue NF. The structure and function of telomerase reverse transcriptase. Annu Rev Biochem 2006; 75: 493–517. 16756500 10.1146/annurev.biochem.75.103004.142412

[bib4] Blackburn EH. Switching and signaling at the telomere. Cell 2001; 106: 661–673. 11572773 10.1016/s0092-8674(01)00492-5

[bib5] Jaskelioff M, Muller FL, Paik JH, Thomas E, Jiang S, Adams AC et al. Telomerase reactivation reverses tissue degeneration in aged telomerase-deficient mice. Nature 2011; 469: 102–106. 21113150 10.1038/nature09603 PMC3057569

[bib6] Rudolph KL, Chang S, Lee HW, Blasco M, Gottlieb GJ, Greider C et al. Longevity, stress response, and cancer in aging telomerase-deficient mice. Cell 1999; 96: 701–712. 10089885 10.1016/s0092-8674(00)80580-2

[bib7] Harley CB. Telomerase and cancer therapeutics. Nat Rev Cancer 2008; 8: 167–179. 18256617 10.1038/nrc2275

[bib8] Ornish D, Lin J, Daubenmier J, Weidner G, Epel E, Kemp C et al. Increased telomerase activity and comprehensive lifestyle changes: a pilot study. Lancet Oncol 2008; 9: 1048–1057. 18799354 10.1016/S1470-2045(08)70234-1

[bib9] Kim NW, Piatyszek MA, Prowse KR, Harley CB, West MD, Ho PL et al. Specific association of human telomerase activity with immortal cells and cancer. Science 1994; 266: 2011–2015. 7605428 10.1126/science.7605428

[bib10] Lee HW, Blasco MA, Gottlieb GJ, Horner JW 2nd, Greider CW, DePinho RA. Essential role of mouse telomerase in highly proliferative organs. Nature 1998; 392: 569–574. 9560153 10.1038/33345

[bib11] Epel ES, Blackburn EH, Lin J, Dhabhar FS, Adler NE, Morrow JD et al. Accelerated telomere shortening in response to life stress. Proc Natl Acad Sci USA 2004; 101: 17312–17315. 15574496 10.1073/pnas.0407162101 PMC534658

[bib12] Simon NM, Smoller JW, McNamara KL, Maser RS, Zalta AK, Pollack MH et al. Telomere shortening and mood disorders: preliminary support for a chronic stress model of accelerated aging. Biol Psychiatry 2006; 60: 432–435. 16581033 10.1016/j.biopsych.2006.02.004

[bib13] Kao HT, Cawthon RM, Delisi LE, Bertisch HC, Ji F, Gordon D et al. Rapid telomere erosion in schizophrenia. Mol Psychiatry 2008; 13: 118–119. 18202693 10.1038/sj.mp.4002105

[bib14] Lee J, Jo YS, Sung YH, Hwang IK, Kim H, Kim SY et al. Telomerase deficiency affects normal brain functions in mice. Neurochem Res 2010; 35: 211–218. 19685288 10.1007/s11064-009-0044-3

[bib15] Zhou QG, Hu Y, Wu DL, Zhu LJ, Chen C, Jin X et al. Hippocampal telomerase is involved in the modulation of depressive behaviors. J Neurosci 2011; 31: 12258–12269. 21865469 10.1523/JNEUROSCI.0805-11.2011 PMC6623221

[bib16] Yuan XM, Ishibashi S, Hatakeyama S, Saito M, Nakayama J, Nikaido R et al. Presence of telomeric G-strand tails in the telomerase catalytic subunit TERT knockout mice. Genes Cells 1999; 4: 563–572. 10583505 10.1046/j.1365-2443.1999.00284.x

[bib17] Suh H, Consiglio A, Ray J, Sawai T, D'Amour KA, Gage FH. *In vivo* fate analysis reveals the multipotent and self-renewal capacities of Sox2+ neural stem cells in the adult hippocampus. Cell Stem Cell 2007; 1: 515–528. 18371391 10.1016/j.stem.2007.09.002 PMC2185820

[bib18] Fejgin K, Palsson E, Wass C, Svensson L, Klamer D. Nitric oxide signaling in the medial prefrontal cortex is involved in the biochemical and behavioral effects of phencyclidine. Neuropsychopharmacology 2008; 33: 1874–1883. 17895915 10.1038/sj.npp.1301587

[bib19] Zhou QG, Zhu LJ, Chen C, Wu HY, Luo CX, Chang L et al. Hippocampal neuronal nitric oxide synthase mediates the stress-related depressive behaviors of glucocorticoids by downregulating glucocorticoid receptor. J Neurosci 2011; 31: 7579–7590. 21613472 10.1523/JNEUROSCI.0004-11.2011 PMC6633122

[bib20] Nelson RJ, Demas GE, Huang PL, Fishman MC, Dawson VL, Dawson TM et al. Behavioural abnormalities in male mice lacking neuronal nitric oxide synthase. Nature 1995; 378: 383–386. 7477374 10.1038/378383a0

[bib21] Kaster MP, Ferreira PK, Santos AR, Rodrigues AL. Effects of potassium channel inhibitors in the forced swimming test: possible involvement of L-arginine-nitric oxide-soluble guanylate cyclase pathway. Behav Brain Res 2005; 165: 204–209. 16122818 10.1016/j.bbr.2005.06.031

[bib22] Zhou QG, Hu Y, Hua Y, Hu M, Luo CX, Han X et al. Neuronal nitric oxide synthase contributes to chronic stress-induced depression by suppressing hippocampal neurogenesis. J Neurochem 2007; 103: 1843–1854. 17854383 10.1111/j.1471-4159.2007.04914.x

[bib23] Alonso R, Griebel G, Pavone G, Stemmelin J, Le Fur G, Soubrie P. Blockade of CRF(1) or V(1b) receptors reverses stress-induced suppression of neurogenesis in a mouse model of depression. Mol Psychiatry 2004; 9: 278–286. 14699428 10.1038/sj.mp.4001464

[bib24] Koolhaas JM, Coppens CM, de Boer SF, Buwalda B, Meerlo P, Timmermans PJ. The resident–intruder paradigm: a standardized test for aggression, violence and social stress. J Vis Exp 2013; 77: e4367. 23852258 10.3791/4367 PMC3731199

[bib25] Zhang J, Huang XY, Ye ML, Luo CX, Wu HY, Hu Y et al. Neuronal nitric oxide synthase alteration accounts for the role of 5-HT1A receptor in modulating anxiety-related behaviors. J Neurosci 2010; 30: 2433–2441. 20164327 10.1523/JNEUROSCI.5880-09.2010 PMC6634557

[bib26] Griebel G, Simiand J, Serradeil-Le Gal C, Wagnon J, Pascal M, Scatton B et al. Anxiolytic- and antidepressant-like effects of the non-peptide vasopressin V1b receptor antagonist, SSR149415, suggest an innovative approach for the treatment of stress-related disorders. Proc Natl Acad Sci USA 2002; 99: 6370–6375. 11959912 10.1073/pnas.092012099 PMC122955

[bib27] Russo SJ, Nestler EJ. The brain reward circuitry in mood disorders. Nat Rev Neurosci 2013; 14: 609–625. 23942470 10.1038/nrn3381 PMC3867253

[bib28] Drevets WC, Price JL, Furey ML. Brain structural and functional abnormalities in mood disorders: implications for neurocircuitry models of depression. Brain Struct Funct 2008; 213: 93–118. 18704495 10.1007/s00429-008-0189-x PMC2522333

[bib29] Dhir A, Kulkarni SK. Nitric oxide and major depression. Nitric Oxide 2011; 24: 125–131. 21335097 10.1016/j.niox.2011.02.002

[bib30] Berton O, Nestler EJ. New approaches to antidepressant drug discovery: beyond monoamines. Nat Rev Neurosci 2006; 7: 137–151. 16429123 10.1038/nrn1846

[bib31] Savitz J, Lucki I, Drevets WC. 5-HT(1A) receptor function in major depressive disorder. Prog Neurobiol 2009; 88: 17–31. 19428959 10.1016/j.pneurobio.2009.01.009 PMC2736801

[bib32] Alekseyenko OV, Kravitz EA. Serotonin and the search for the anatomical substrate of aggression. Fly 2014; 8: 200–205. 25923771 10.1080/19336934.2015.1045171 PMC4594415

[bib33] Popova NK, Naumenko VS. 5-HT1A receptor as a key player in the brain 5-HT system. Rev Neurosci 2013; 24: 191–204. 23492554 10.1515/revneuro-2012-0082

[bib34] Popova NK, Naumenko VS, Plyusnina IZ. Involvement of brain serotonin 5-HT1A receptors in genetic predisposition to aggressive behavior. Neurosci Behav Physiol 2007; 37: 631–635. 17657435 10.1007/s11055-007-0062-z

[bib35] Tyrka AR, Carpenter LL, Kao HT, Porton B, Philip NS, Ridout SJ et al. Association of telomere length and mitochondrial DNA copy number in a community sample of healthy adults. Exp Gerontol 2015; 66: 17–20. 25845980 10.1016/j.exger.2015.04.002 PMC4459604

[bib36] Drury SS, Theall K, Gleason MM, Smyke AT, De Vivo I, Wong JYY et al. Telomere length and early severe social deprivation: linking early adversity and cellular aging. Mol Psychiatry 2012; 17: 719–727. 21577215 10.1038/mp.2011.53 PMC3518061

[bib37] Entringer S, Epel ES, Kumsta R, Lin J, Hellhammer DH, Blackburn EH et al. Stress exposure in intrauterine life is associated with shorter telomere length in young adulthood. Proc Natl Acad Sci USA 2011; 108: E513–E518. 21813766 10.1073/pnas.1107759108 PMC3158153

[bib38] Wei YB, Backlund L, Wegener G, Mathe AA, Lavebratt C. Telomerase dysregulation in the hippocampus of a rat model of depression: normalization by lithium. Int J Neuropsychopharmacol 2015; 18: pyv002. 25618407 10.1093/ijnp/pyv002 PMC4540104

[bib39] Gross C, Zhuang X, Stark K, Ramboz S, Oosting R, Kirby L et al. Serotonin1A receptor acts during development to establish normal anxiety-like behaviour in the adult. Nature 2002; 416: 396–400. 11919622 10.1038/416396a

[bib40] Santarelli L, Saxe M, Gross C, Surget A, Battaglia F, Dulawa S et al. Requirement of hippocampal neurogenesis for the behavioral effects of antidepressants. Science 2003; 301: 805–809. 12907793 10.1126/science.1083328

[bib41] van Erp AMM, Miczek KA. Aggressive behavior, increased accumbal dopamine, and decreased cortical serotonin in rats. J Neurosci 2000; 20: 9320–9325. 11125011 10.1523/JNEUROSCI.20-24-09320.2000 PMC6773005

[bib42] Popova NK, Avgustinovich DF, Kolpakov VG, Plyusnina IZ. Specific [H-3]8-OH-DPAT binding in brain regions of rats genetically predisposed to various defense behavior strategies. Pharmacol Biochem Behav 1998; 59: 793–797. 9586833 10.1016/s0091-3057(97)00504-2

[bib43] Olivier B, Mos J, vanOorschot R, Hen R. Serotonin receptors and animal models of aggressive behavior. Pharmacopsychiatry 1995; 28: 80–90. 10.1055/s-2007-979624 8614705

[bib44] Pruss K, Skrebuhhova-Malmros T, Rudissaar R, Matto V, Allikmets L. 5-HT1A receptor agonists buspirone and gepirone attenuate apomorphine-induced aggressive behaviour in adult male Wistar rats. J Physiol Pharmacol 2000; 51: 833–846. 11220492

[bib45] Eitan E, Hutchison ER, Mattson MP. Telomere shortening in neurological disorders: an abundance of unanswered questions. Trends Neurosci 2014; 37: 256–263. 24698125 10.1016/j.tins.2014.02.010 PMC4008659

[bib46] Borroni B, Costanzi C, Padovani A. Genetic susceptibility to behavioural and psychological symptoms in Alzheimer disease. Curr Alzheimer Res 2010; 7: 158–164. 19715553 10.2174/156720510790691173

[bib47] Verhoeven JE, Revesz D, Epel ES, Lin J, Wolkowitz OM, Penninx BW. Major depressive disorder and accelerated cellular aging: results from a large psychiatric cohort study. Mol Psychiatry 2014; 19: 895–901. 24217256 10.1038/mp.2013.151

[bib48] Tomas-Loba A, Flores I, Fernandez-Marcos PJ, Cayuela ML, Maraver A, Tejera A et al. Telomerase reverse transcriptase delays aging in cancer-resistant mice. Cell 2008; 135: 609–622. 19013273 10.1016/j.cell.2008.09.034

[bib49] Li Y, Tergaonkar V. Noncanonical functions of telomerase: implications in telomerase-targeted cancer therapies. Cancer Res 2014; 74: 1639–1644. 24599132 10.1158/0008-5472.CAN-13-3568

[bib50] Park JI, Venteicher AS, Hong JY, Choi J, Jun S, Shkreli M et al. Telomerase modulates Wnt signalling by association with target gene chromatin. Nature 2009; 460: 66–72. 19571879 10.1038/nature08137 PMC4349391

