# Supplementary material for: Reactivation of Tert in the medial prefrontal cortex and hippocampus rescues aggression and depression of Tert−/− mice
Source: Transl Psychiatry. 2016 Jun 14;6(6):e836–. doi: 10.1038/tp.2016.106 (PMC4931604; doi:10.1038/tp.2016.106)
Supplement: Supplementary Figure Legends [file tp2016106x1.doc]

**Supplementary figure legends**

**Supplementary Figure 1. Re-expression of *Tert* in the mPFC reversed the aggressive behavior of *Tert^-/-^* mice.**

(a, b) Offensive aggressive behavior: the latency to the first attack bite (left), the total number of attacks (middle), and the total duration of attack episodes (right) showed the rescue of the aggressive phenotype of *Tert^-/-^* mice by re-expression of *Tert* in the mPFC (b) but not in the hippocampus (a) (n = 20 each group). Data are mean ± SEM. *, p < 0.05; **, p < 0.01; ***, p < 0.001 compared with WT/LV-GFP; ^#^, p < 0.05; ^##^, p < 0.01 compared with *Tert*^-/-^/LV-GFP; ns, no significance compared with *Tert*^-/-^/LV-GFP; one-way ANOVA.

**Supplementary Figure 2**. **Effective expression of TERT by LV-*mTert*-EGFP.**

LV-*mTert*-EGFP or LV-EGFP was injected into the DG or mPFC of WT or *Tert^-/-^* mice. Western blots showed EGFP and FLAG-tagged TERT expression in the hippocampus (a-d) and mPFC (e-f) of WT or *Tert^-/-^* mice. The comparable amount of EGFP expression across mice indicates a similar level of viral infection by LV-*mTert*-EGFP or LV-EGFP into the hippocampus and mPFC. FLAG was used to indirectly detect the expression of TERT: 3X FLAG (2.75 kDa) was fused to TERT (127 kDa). GAPDH was used as a loading control.

**Supplementary Figure 3. Distribution of cells infected with virus in the DG and mPFC.**

(a) Representative images of virus-infected brain showed a wider spread of virus in the mPFC. (b) The level of infectivity of the virus was semi-quantified on the brain atlas. Green dots represent approximately 5 EGFP-expressing cells in the corresponding areas of the mPFC. (c) Representative images of virus-infected brain showed the coverage of virus in the hippocampus. (d) The viral spread and infectivity were semi-quantified on the brain atlas. Green dots represent approximately 5 EGFP-expressing cells in the corresponding areas of the hippocampus.

**Supplementary Figure 4**. **Neuronal expression of 5-HTR1A and nNOS in the DG and mPFC.**

(a-b) Representative images show co-localization of 5-HTR1A with a neuronal marker, NeuN, but not the astrocyte-specific marker GFAP in the DG and mPFC. Arrowheads indicate NeuN^+^ neurons that co-express 5-HTR1A. (c-d) Representative images show that nNOS-expressing cells are NeuN^+^ neurons but not GFAP^+^ astrocytes in the DG and mPFC. Note that nNOS^+^ cells in the hippocampus are located in the hilus. Arrowheads indicate NeuN^+^ neurons that also express nNOS. The area defined by a white box in the top panel is magnified in the bottom panel to display the identity of 5-HTR1A- or nNOS-expressing cells.

**Supplementary Figure 5. The concentration of 5-HT and NO in the hippocampus and mPFC of *Tert*^-/-^ mice.**

(**a**) The 5-HT concentration in the hippocampus and mPFC in *Tert^-/-^* and WT mice. Hippocampus: WT, n = 8; *Tert^-/-^* mice, n = 8; mPFC: WT, n = 8; *Tert^-/-^* mice, n = 8. (**b**) The measurement of NO concentration in the hippocampus and mPFC in *Tert^-/-^* and WT mice. Hippocampus: WT, n = 6; *Tert^-/-^* mice, n = 7; mPFC: WT, n = 7; *Tert^-/-^* mice, n = 6. Data are mean ± SEM. **, p < 0.01, t-test, compared with WT mice.
