# Supplementary figures and images for: Reactivation of Tert in the medial prefrontal cortex and hippocampus rescues aggression and depression of Tert−/− mice
Source: Transl Psychiatry. 2016 Jun 14;6(6):e836–. doi: 10.1038/tp.2016.106 (PMC4931604; doi:10.1038/tp.2016.106)

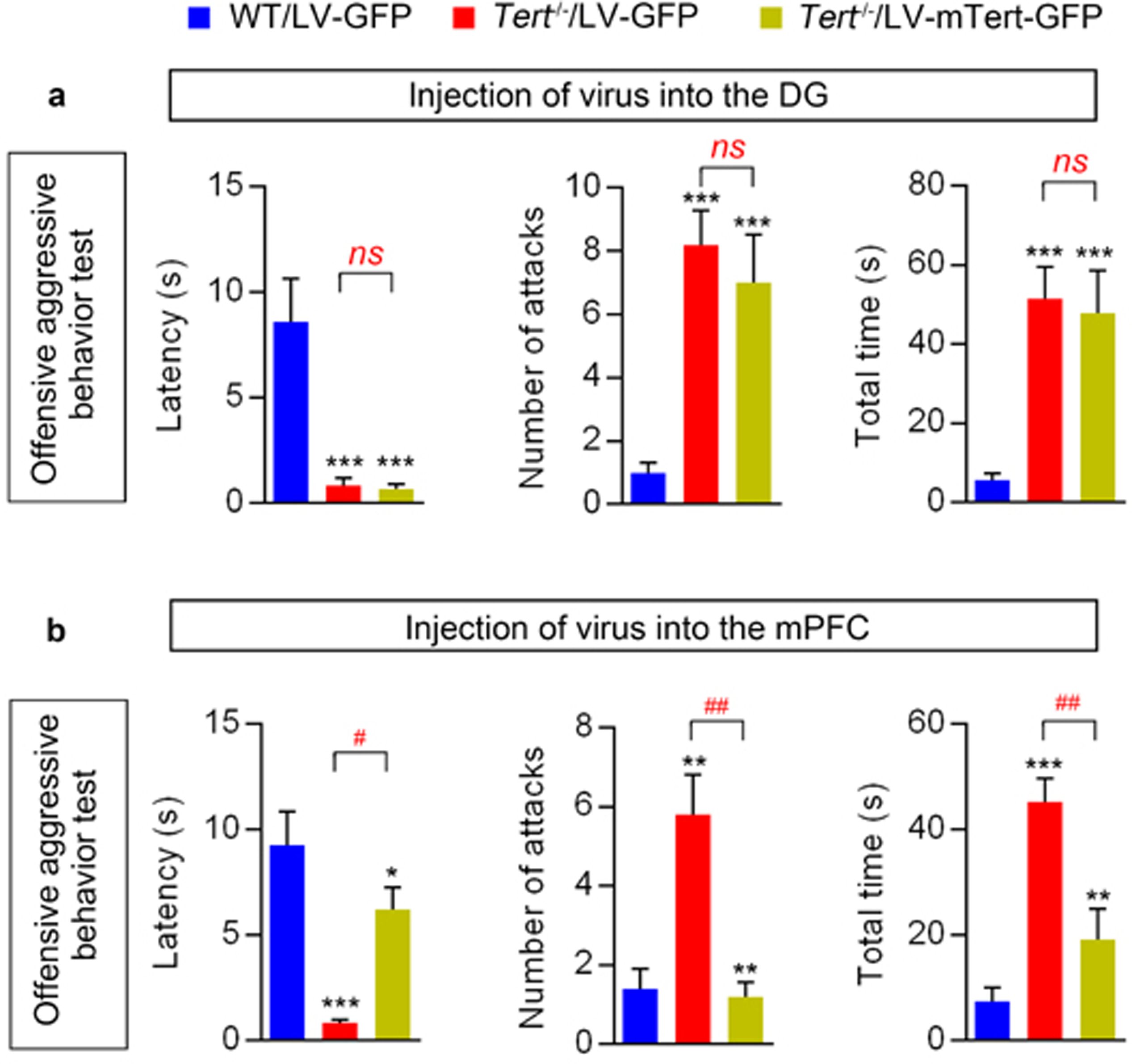

Supplement: Supplementary Figure 1 [file tp2016106x2.tif]

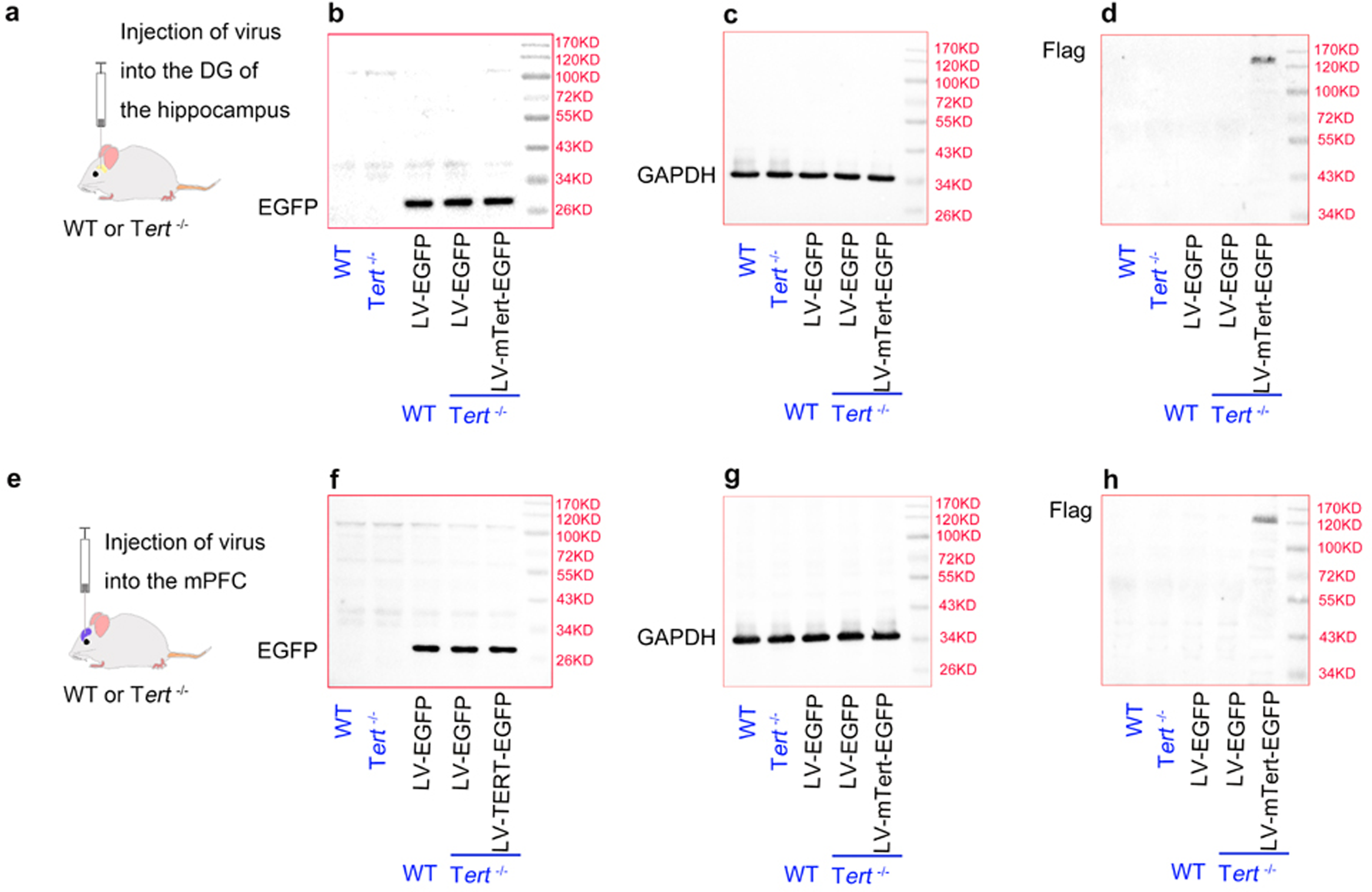

Supplement: Supplementary Figure 2 [file tp2016106x3.tif]

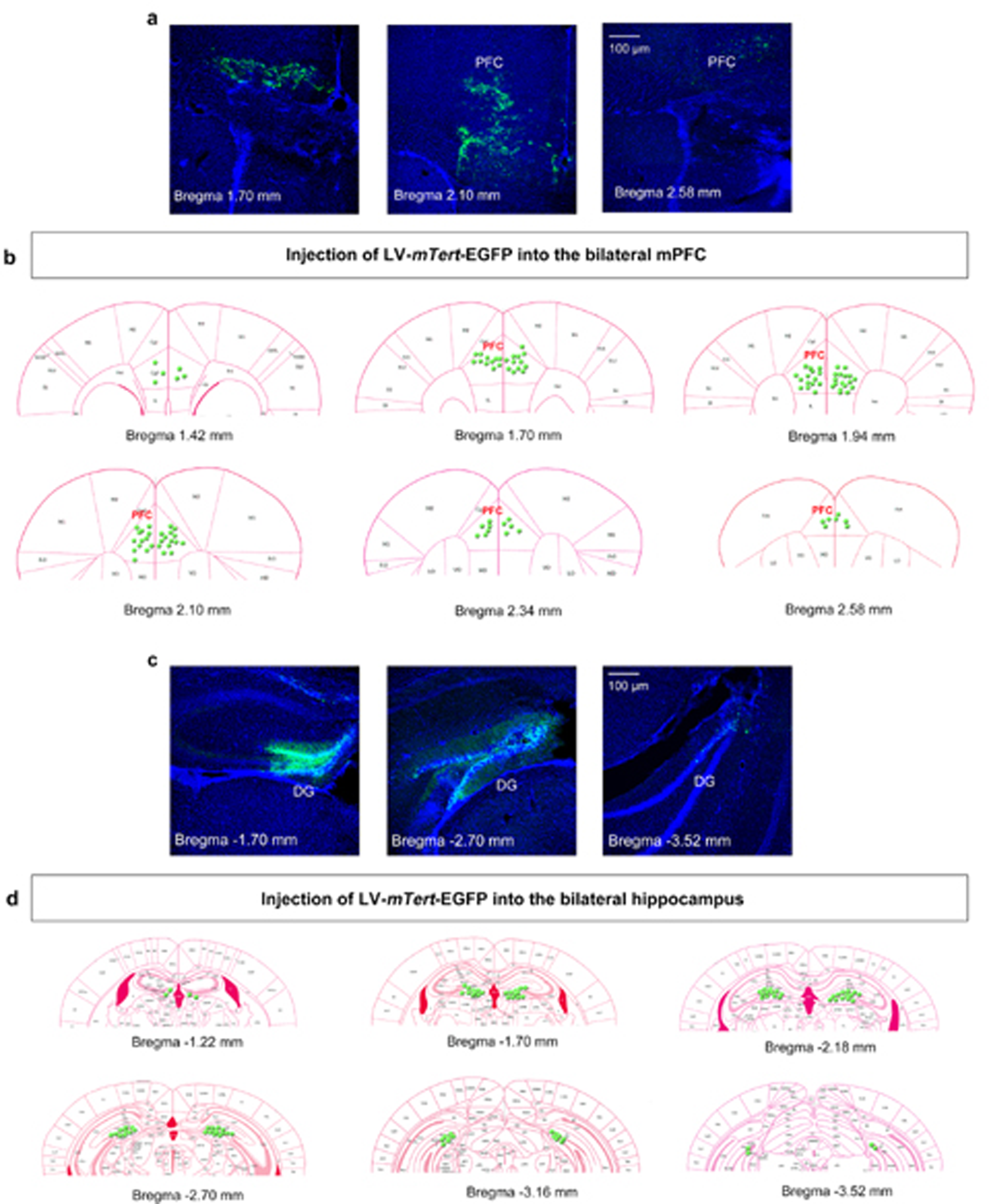

Supplement: Supplementary Figure 3 [file tp2016106x4.tif]

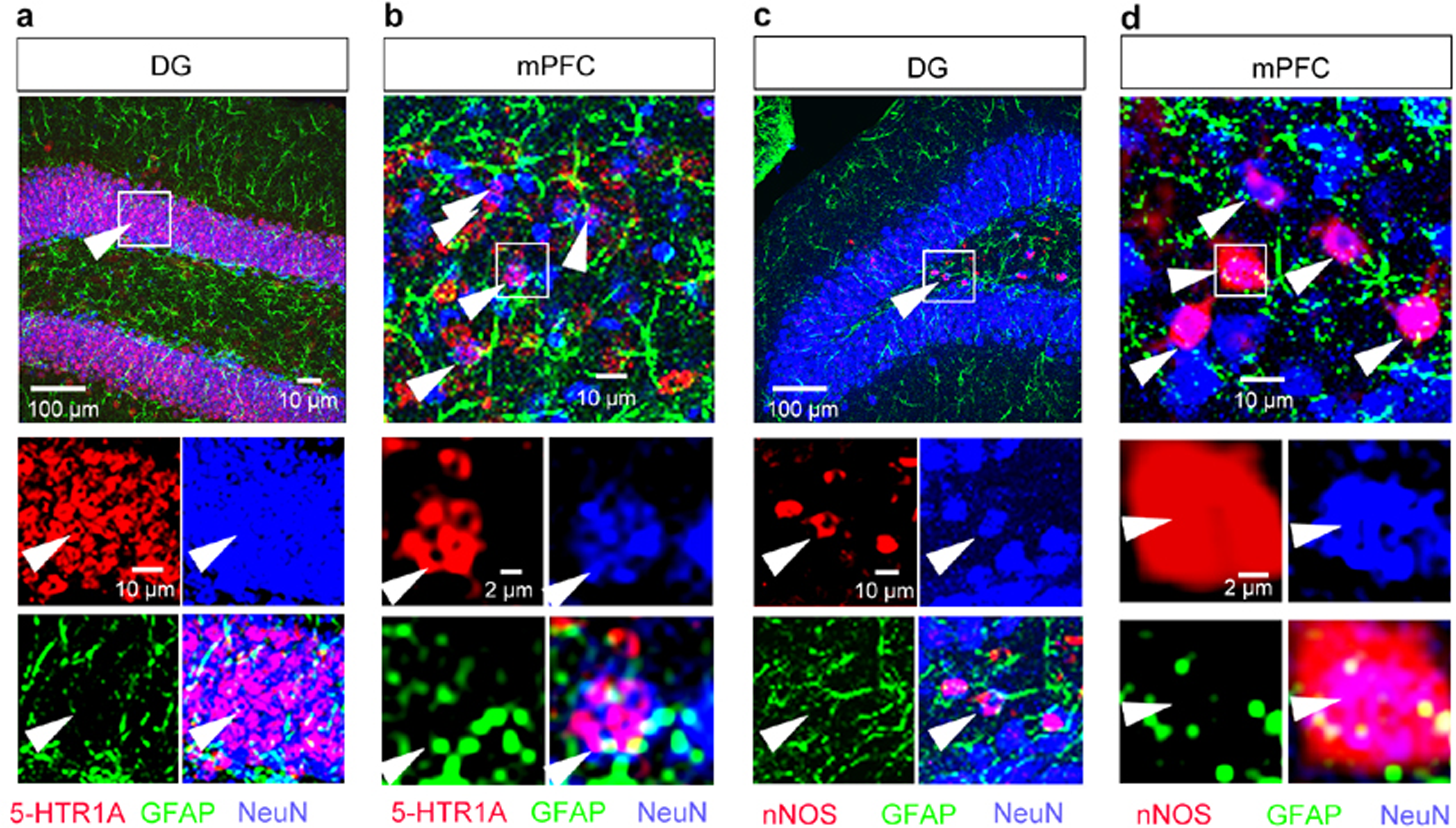

Supplement: Supplementary Figure 4 [file tp2016106x5.tif]

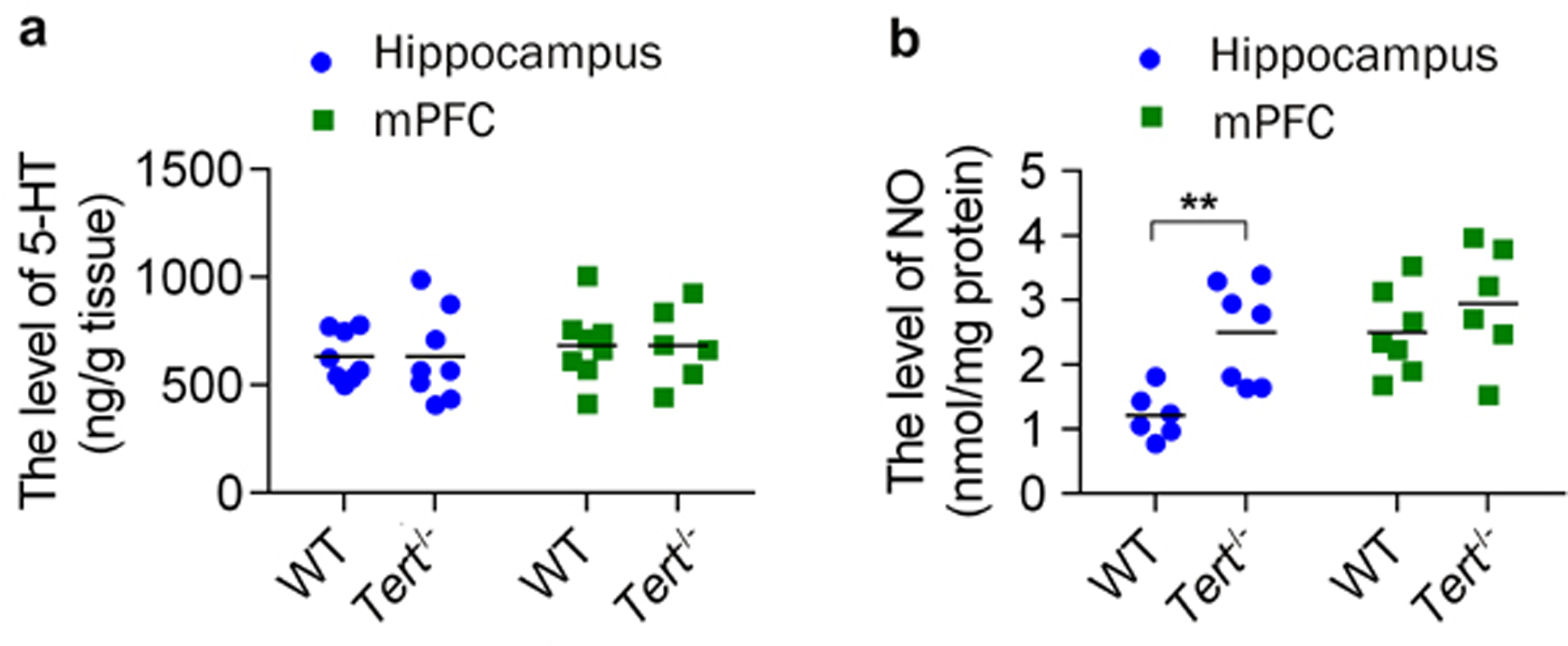

Supplement: Supplementary Figure 5 [file tp2016106x6.tif]
