# Supplementary material for: Reactivation of Tert in the medial prefrontal cortex and hippocampus rescues aggression and depression of Tert−/− mice
Source: Transl Psychiatry. 2016 Jun 14;6(6):e836–. doi: 10.1038/tp.2016.106 (PMC4931604; doi:10.1038/tp.2016.106)
Supplement: Supplementary Table 1 [file tp2016106x7.doc]

| Resident-intruder  (fig 2b) | Latency to the first attack | One-way ANOVA, F_2, 12_ = 13.56, P = 0.0008  WT/LV-GFP vs. *Tert* ^-/-^/LV-GFP, P = 0.0021;  *Tert* ^-/-^/LV-GFP vs. *Tert* ^-/-^/LV-mTERT-GFP, P = 0.9941 | WT/LV-GFP  n = 20;  *Tert* ^-/-^/LV-GFP  n = 20;  *Tert* ^-/-^/LV-mTERT-GFP  n = 20; |
| --- | --- | --- | --- |
|  | # of attacks | One-way ANOVA, F_2, 12_ = 12.61, P = 0.0011  WT/LV-GFP vs. *Tert* ^-/-^/LV-GFP, P = 0.0014;  *Tert* ^-/-^/LV-GFP vs. *Tert* ^-/-^/LV-mTERT-GFP, P = 0.7212 |  |
|  | Total time of attacks | One-way ANOVA, F_2, 12_ = 11.00, P = 0.0019  WT/LV-GFP vs. *Tert* ^-/-^/LV-GFP, P = 0.0032;  *Tert* ^-/-^/LV-GFP vs. *Tert* ^-/-^/LV-mTERT-GFP, P = 0.9420 |  |
| TST  (fig 2c) | Immobility | One-way ANOVA, F_2, 54_ = 8.472, P = 0.0006  WT/LV-GFP vs. *Tert* ^-/-^/LV-GFP, P = 0.0017;  *Tert* ^-/-^/LV-GFP vs. *Tert* ^-/-^/LV-mTERT-GFP, P = 0.0022 | WT/LV-GFP  n = 20;  *Tert* ^-/-^/LV-GFP  n = 19;  *Tert* ^-/-^/LV-mTERT-GFP  n = 18; |
| FST  (fig 2c) | Immobility | One-way ANOVA, F_2, 48_ = 6.824, P = 0.0025  WT/LV-GFP vs. mTERT^-/-^/LV-GFP, P = 0.0143;  *Tert* ^-/-^/LV-GFP vs. *Tert* ^-/-^/LV-mTERT-GFP, P = 0.0033 | WT/LV-GFP  n= 18;  *Tert* ^-/-^/LV-GFP  n= 16;  *Tert* ^-/-^/LV-mTERT-GFP  n= 17; |
| EMT  (fig 2c) | Time to entry to open arm | ANOVA, F_2, 48_ = 5.063, P = 0.0101  WT/LV-GFP vs. *Tert* ^-/-^/LV-GFP, P = 0.0202;  *Tert* ^-/-^/LV-GFP vs. *Tert* ^-/-^/LV-mTERT-GFP, P = 0.0242 | WT/LV-GFP  n = 18;  *Tert* ^-/-^/LV-GFP  n = 17;  *Tert* ^-/-^/LV-mTERT-GFP  n = 16; |
| Resident-intruder  (fig 2d) | Latency to the first attack | One-way ANOVA, F_2, 12_ = 9.598, P = 0.0032  WT/LV-GFP vs. *Tert*^-/-^/LV-GFP, P = 0.0123;  *Tert* ^-/-^/LV-GFP vs. *Tert* ^-/-^/LV-mTERT-GFP, P = 0.0042 | WT/LV-GFP  n = 20;  *Tert* ^-/-^/LV-GFP  n = 20;  *Tert* ^-/-^/LV-mTERT-GFP  n = 20; |
|  | # of attacks | One-way ANOVA, F_2, 12_ = 23.89, P = 0.0001  WT/LV-GFP vs. *Tert* ^-/-^/LV-GFP, P = 0.0001;  *Tert* ^-/-^/LV-GFP vs. *Tert* ^-/-^/LV-mTERT-GFP, P = 0.0070 |  |
|  | Total time of attacks | One-way ANOVA, F_2, 12_ = 25.57, P = 0.0001  WT/LV-GFP vs. *Tert* ^-/-^/LV-GFP, P = 0.0001;  *Tert* ^-/-^/LV-GFP vs. *Tert* ^-/-^/LV-mTERT-GFP, P = 0.0002 |  |
| TST  (fig 2e) | Immobility | One-way ANOVA, F_2, 53_ = 16.61, P = 0.0001  WT/LV-GFP vs. *Tert* ^-/-^/LV-GFP, P = 0.0017;  *Tert* ^-/-^/LV-GFP vs. *Tert* ^-/-^/LV-mTERT-GFP, P = 0.7953 | WT/LV-GFP  n = 20;  *Tert* ^-/-^/LV-GFP  n = 17;  *Tert* ^-/-^/LV-mTERT-GFP  n = 19; |
| FST  (fig 2e) | Immobility | One-way ANOVA, F_2, 50_ = 9.153, P = 0.0004  WT/LV-GFP vs. *Tert* ^-/-^/LV-GFP, P = 0.0005;  *Tert* ^-/-^/LV-GFP vs. *Tert* ^-/-^/LV-mTERT-GFP, P = 0.6551 | WT/LV-GFP  n = 18;  *Tert* ^-/-^/LV-GFP  n = 17;  *Tert* ^-/-^/LV-mTERT-GFP  n = 18; |
| EMT  (Fig 2e) | Time to entry to open arm | One-way ANOVA, F_2, 52_ = 10.51, P = 0.0001;  WT/LV-GFP vs. *Tert* ^-/-^/LV-GFP, P = 0.0004  *Tert* ^-/-^/LV-GFP vs. *Tert* ^-/-^/LV-mTERT-GFP, P = 0.9478 | WT/LV-GFP  n = 20;  *Tert* ^-/-^/LV-GFP  n = 19;  *Tert* ^-/-^/LV-mTERT-GFP  n = 16; |

Table 1) Summary of Statistics for Figure 2
