# Supplementary material for: Reactivation of Tert in the medial prefrontal cortex and hippocampus rescues aggression and depression of Tert−/− mice
Source: Transl Psychiatry. 2016 Jun 14;6(6):e836–. doi: 10.1038/tp.2016.106 (PMC4931604; doi:10.1038/tp.2016.106)
Supplement: Supplementary Table 2 [file tp2016106x8.doc]

| Resident-intruder  (fig 4b) | Latency to the first attack | One-way ANOVA, F_3, 16_ = 49.79, P = 0.0001  *Tert* ^-/-^/DMSO vs. *Tert* ^-/-^/8-OH-DPAT, P = 0.9969  *Tert* ^-/-^/DMSO vs. *Tert* ^-/-^/7-NI, P = 0.2340 | WT/DMSO  n = 20;  *Tert* ^-/-^/DMSO  n = 20;  *Tert* ^-/-^/7-NI  n = 20;  *Tert* ^-/-^/8-OH-DPAT  n = 20; |
| --- | --- | --- | --- |
|  | # of attacks | One-way ANOVA, F_3, 16_ = 13.34, P = 0.0001  *Tert* ^-/-^/DMSO vs. *Tert* ^-/-^/8-OH-DPAT, P = 0.2440;  *Tert* ^-/-^/DMSO vs. *Tert* ^-/-^/7-NI, P = 0.1523 |  |
|  | Total time of attacks | One-way ANOVA, F_3, 16_ = 19.93, P = 0.0001  *Tert* ^-/-^/DMSO vs. *Tert* ^-/-^/8-OH-DPAT, P = 0.9982;  *Tert* ^-/-^/DMSO vs. *Tert* ^-/-^/7-NI, P = 0.8688 |  |
| TST  (fig 4c) | Immobility | One-way ANOVA, F_3, 42_ = 7.411, P = 0.0004  *Tert* ^-/-^/DMSO vs. *Tert* ^-/-^/7-NI, P = 0.0004;  *Tert* ^-/-^/DMSO vs. *Tert* ^-/-^/8-OH-DPAT, P = 0.0057 | WT/DMSO  n = 15;  *Tert* ^-/-^/DMSO  n = 15;  *Tert* ^-/-^/7-NI  n = 10;  *Tert* ^-/-^/8-OH-DPAT  n = 10; |
| FST  (fig 4c) | Immobility | One-way ANOVA, F_3, 46_ = 11.88, P = 0.0001  *Tert* ^-/-^/DMSO vs. *Tert* ^-/-^/7-NI, P = 0.0001;  *Tert* ^-/-^/DMSO vs. *Tert* ^-/-^/8-OH-DPAT, P = 0.0001 | WT/DMSO  n = 15;  *Tert* ^-/-^/DMSO  n = 11;  *Tert* ^-/-^/7-NI  n = 10;  *Tert* ^-/-^/8-OH-DPAT  n = 10; |
| EMT  (fig 4c) | Time to entry to open arm | ANOVA, F_3, 42_ = 13.38, P = 0.0001  *Tert* ^-/-^/DMSO vs. *Tert* ^-/-^/7-NI, P = 0.0002;  *Tert* ^-/-^/DMSO vs. *Tert* ^-/-^/8-OH-DPAT, P = 0.0001 | WT/DMSO  n = 15;  *Tert* ^-/-^/DMSO  n = 11;  *Tert* ^-/-^/7-NI  n = 10;  *Tert* ^-/-^/8-OH-DPAT  n = 10; |
| Resident-intruder  (fig 4c) | Latency to the first attack | One-way ANOVA, F_3, 16_ = 18.38, P = 0.0001  *Tert* ^-/-^/DMSO vs. *Tert* ^-/-^/8-OH-DPAT, P = 0.0002;  *Tert* ^-/-^/DMSO vs. *Tert* ^-/-^/7-NI, P = 0.9983 | WT/DMSO  n = 20;  *Tert* ^-/-^/DMSO  n = 20;  *Tert* ^-/-^/7-NI  n = 20;  *Tert* ^-/-^/8-OH-DPAT  n = 20; |
|  | # of attacks | One-way ANOVA, F_3, 16_ = 17.93, P = 0.0001  *Tert* ^-/-^/DMSO vs. *Tert* ^-/-^/8-OH-DPAT, P = 0.0002;  *Tert* ^-/-^/DMSO vs. *Tert* ^-/-^/7-NI, P = 0.1523 |  |
|  | Total time of attacks | One-way ANOVA, F_3, 16_ = 16.65, P = 0.0001  *Tert* ^-/-^/DMSO vs. *Tert* ^-/-^/8-OH-DPAT, P = 0.0018;  *Tert* ^-/-^/DMSO vs. *Tert* ^-/-^/7-NI, P = 0.8628 |  |
| TST  (fig 4e) | Immobility | One-way ANOVA, F_3, 42_ = 12.21, P = 0.0001  *Tert* ^-/-^/DMSO vs. *Tert* ^-/-^/7-NI, P = 0.8743;  *Tert* ^-/-^/DMSO vs. *Tert* ^-/-^/8-OH-DPAT, P = 0.3500 | WT/DMSO  n = 15;  *Tert* ^-/-^/DMSO  n = 11;  *Tert* ^-/-^/7-NI  n = 10;  *Tert* ^-/-^/8-OH-DPAT  n = 10; |
| FST  (fig 4e) | Immobility | One-way ANOVA, F_3, 42_ = 8.179, P = 0.0002  *Tert* ^-/-^/DMSO vs. *Tert* ^-/-^/7-NI, P = 0.9320;  *Tert* ^-/-^/DMSO vs. *Tert* ^-/-^/8-OH-DPAT, P = 0.4023 | WT/DMSO  n = 15;  *Tert* ^-/-^/DMSO  n = 11;  *Tert* ^-/-^/7-NI  n = 10;  *Tert* ^-/-^/8-OH-DPAT  n = 10; |
| EMT  (Fig 4e) | Time to entry to open arm | One-way ANOVA, F_3, 41_ = 8.396, P = 0.0002  *Tert* ^-/-^/DMSO vs. *Tert* ^-/-^/7-NI, P = 0.9998;  *Tert* ^-/-^/DMSO vs. *Tert* ^-/-^/8-OH-DPAT, P = 0.7333 | WT/DMSO  n = 14;  *Tert* ^-/-^/DMSO  n = 11;  *Tert* ^-/-^/7-NI  n = 10;  *Tert* ^-/-^/8-OH-DPAT  n = 10; |

Table 2) Summary of Statistics for Figure 4
